# Supplementary material for: A highly proton conductive perfluorinated covalent triazine framework via low-temperature synthesis
Source: Nat Commun. 2023 Dec 8;14:8114. doi: 10.1038/s41467-023-43829-4 (PMC10709654; doi:10.1038/s41467-023-43829-4)
Supplement: Supplementary file 1 — Supplementary Information [file 41467_2023_43829_MOESM1_ESM.pdf]

Supplementary Information

A Highly Proton Conductive Perfluorinated Covalent Triazine Framework via Low-Temperature Synthesis

Lijiang Guan[1], Zhaoqi Guo[2], Qi Zhou[1], Jin Zhang[1], Cheng Cheng[1], Shengyao Wang[3], Xiang Zhu[4], Sheng Dai[5], and Shangbin Jin[1]*

[1] School of Chemical Engineering and Technology, Xi’an Jiaotong University, No. 28, Xianning West Road, Xi’an, Shaanxi 710049, China

[2] School of Chemical Engineering, Northwest University, No.229 Taibai North Road, Xi'an, Shaanxi 710069, China

[3] College of Science, Huazhong Agricultural University, Wuhan 430070, China

[4] State Key Laboratory for Oxo Synthesis and Selective Oxidation, Suzhou Research Institute of Lanzhou Institute of Chemical Physics, Chinese Academy of Sciences, Lanzhou 730000, China

[5] Chemical Sciences Division, Oak Ridge National Laboratory, Oak Ridge, TN 37831, USA

*Corresponding author. E-mail: shangbin@xjtu.edu.cn

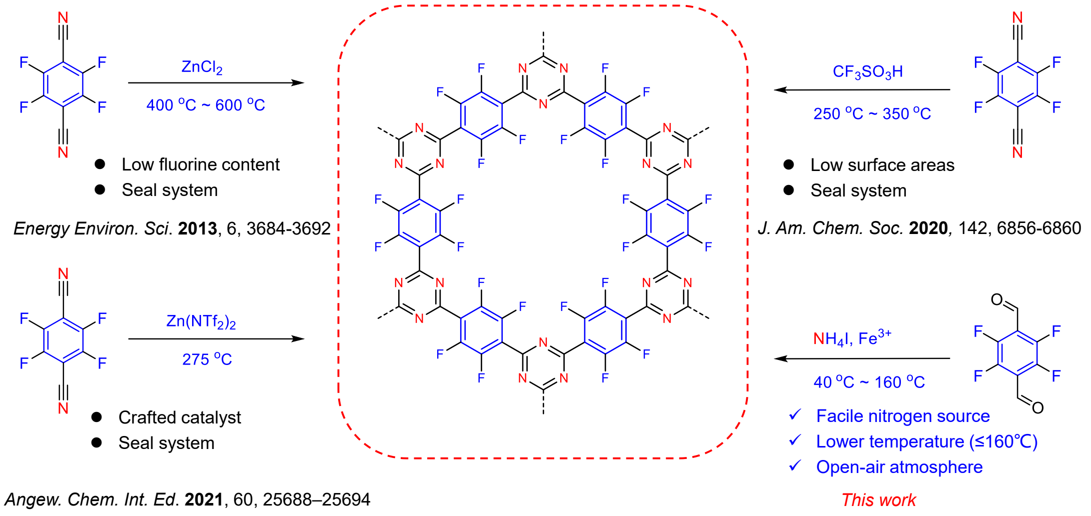

Supplementary Fig. 1. General comparison of previous methods with this work for perfluorinated CTFs.

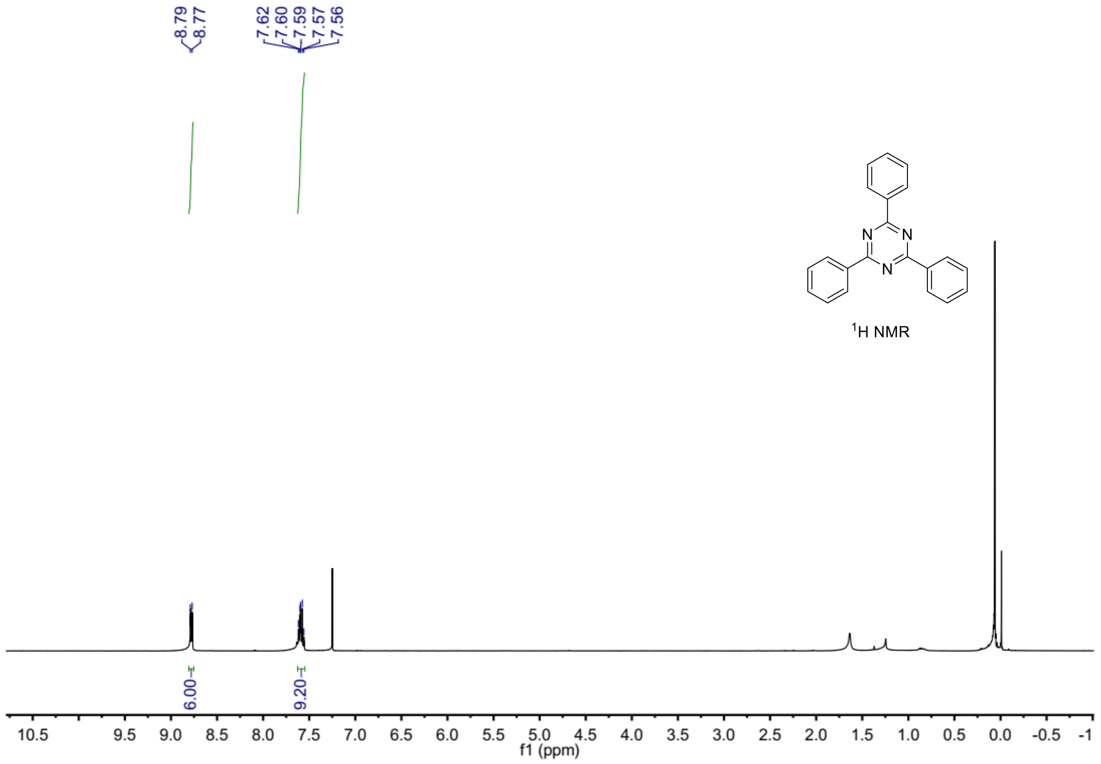

Supplementary Fig. 2. ^{1}H NMR spectra of 2,4,6-triphenyl-1,3,5-triazine in $CDCl_3$.

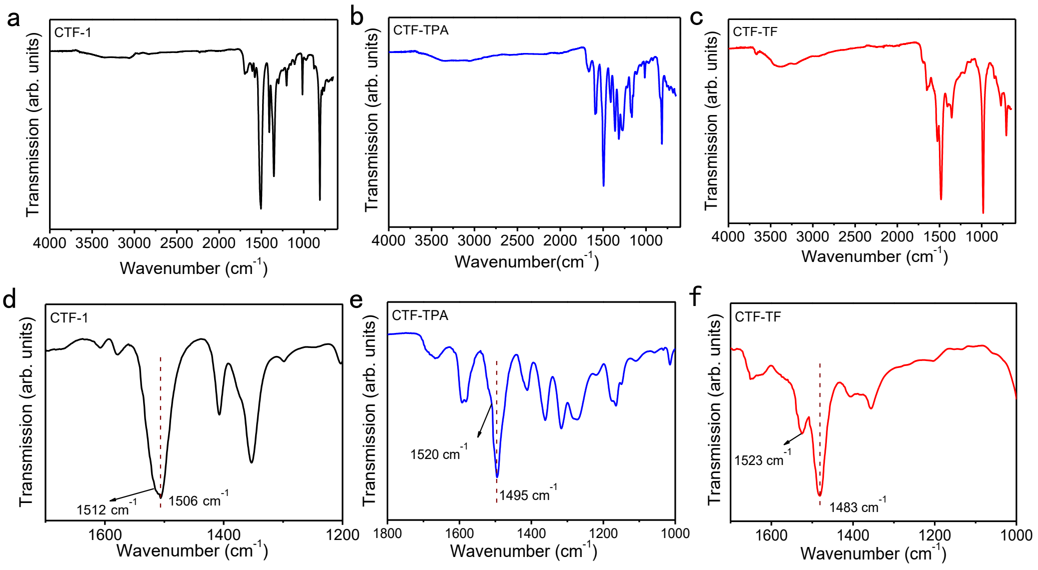

Supplementary Fig. 3. Fourier transform infrared (FT-IR) spectra of (a, d) CTF-1, (b, e) CTF-TPA, (c, f) CTF-TF. C-C stretching in the benzene ring at 1506 cm^{-1} for CTF-1, 1495 cm^{-1} for CTF-TPA as well as 1483 cm^{-1} for CTF-TF.

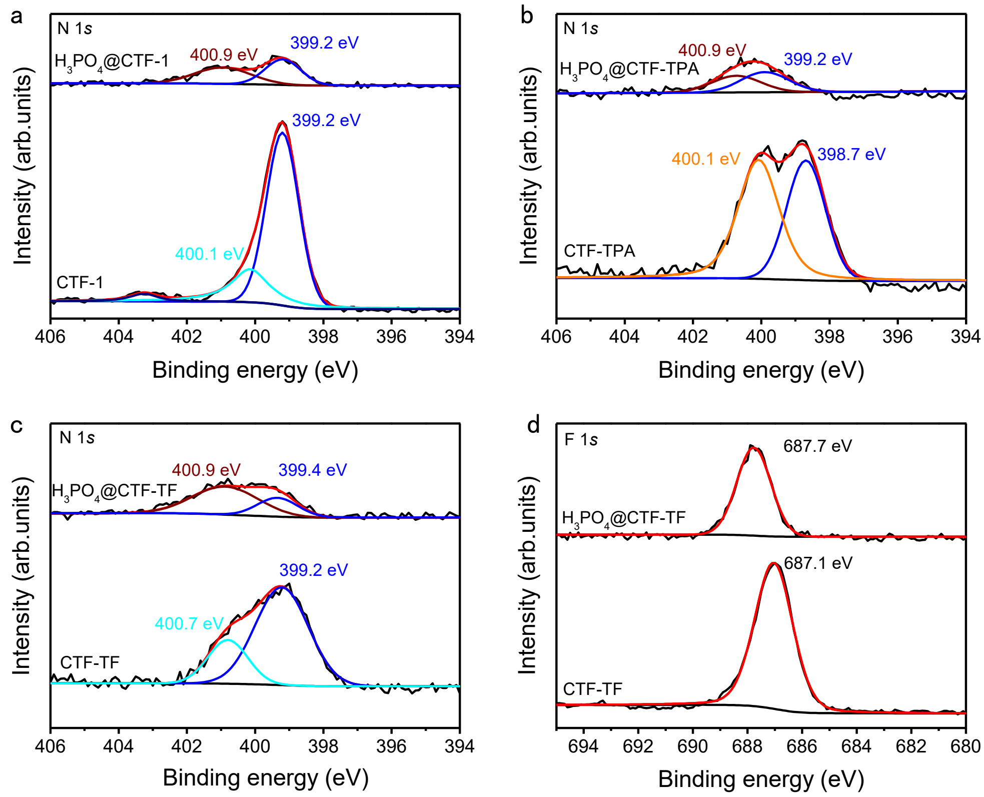

Supplementary Fig. 4. (a) N 1*s* XPS spectra of CTF-1 and H_3PO_4@CTF-1; (b) N 1*s* XPS spectra of CTF-TPA and H_3PO_4@CTF-TPA; (c) N 1*s* XPS spectra of CTF-TF and H_3PO_4@CTF-TF; (d) F 1*s* XPS spectra of CTF-TF and H_3PO_4@CTF-TF.

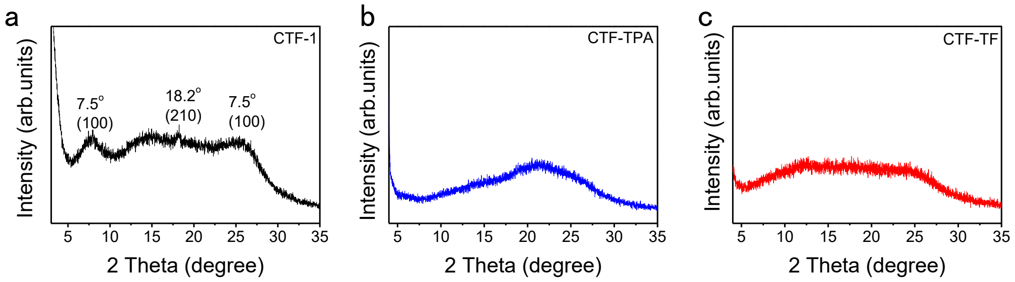

Supplementary Fig. 5. PXRD patterns of (a) CTF-1, (b) CTF-TPA, (c) CTF-TF.

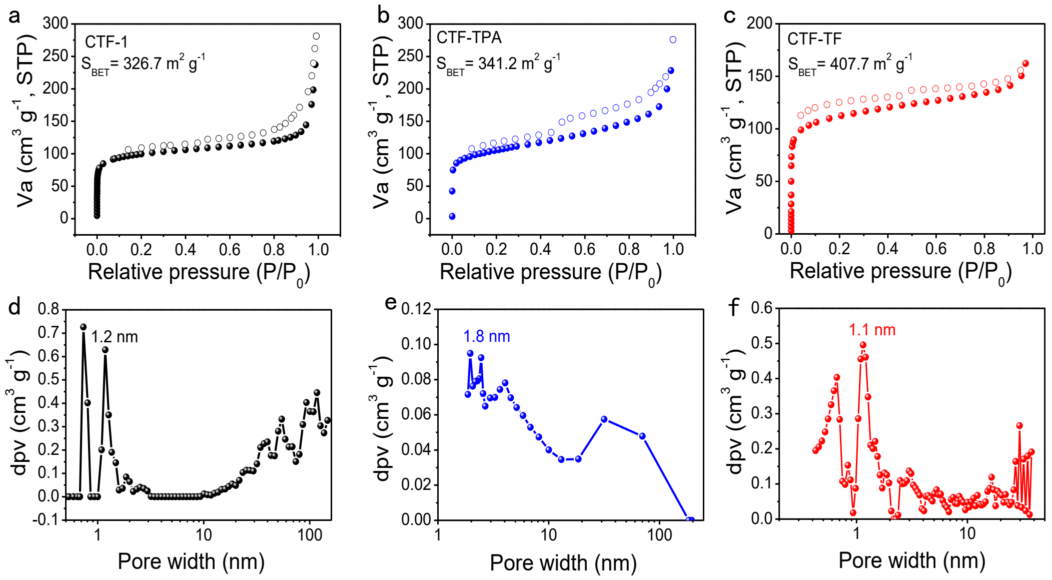

Supplementary Fig. 6. N_2 adsorption (solid circles) and desorption (hollow circles) isotherms (77 K) curves of (a) CTF-1, (b) CTF-TPA and (c) CTF-TF; Pore size distributions of (d) CTF-1, (e) CTF-TPA and (f) CTF-TF.

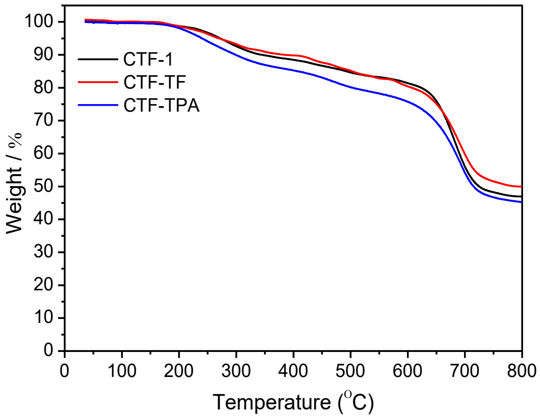

Supplementary Fig. 7. TGA curves of CTFs measured under nitrogen atmosphere.

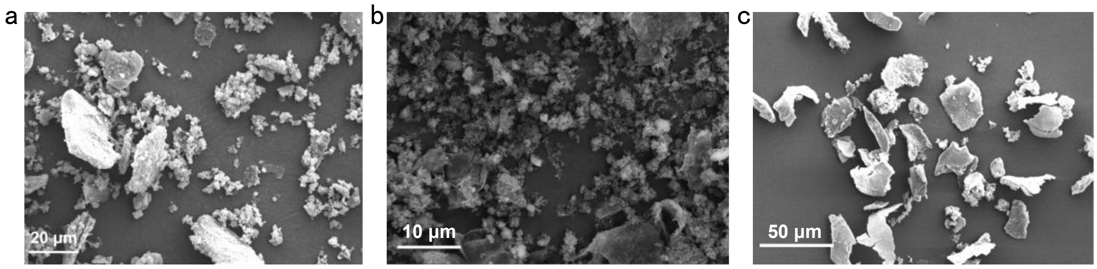

Supplementary Fig. 8. SEM images of (a) CTF-1, (b) CTF-TPA and (c) CTF-TF.

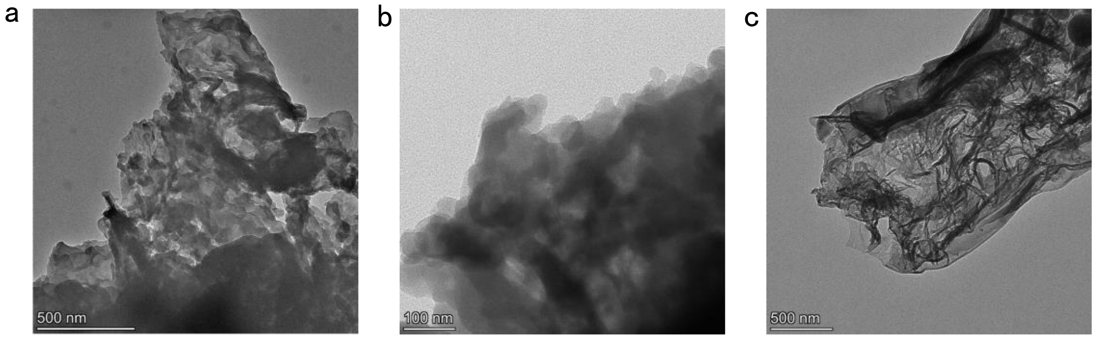

Supplementary Fig. 9. TEM images of (a) CTF-1, (b) CTF-TPA and (c) CTF-TF.

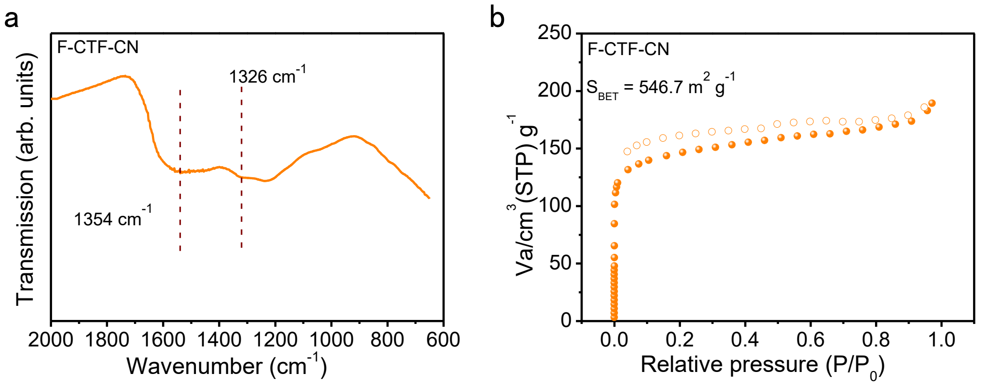

Supplementary Fig. 10. (a) FT-IR spectra of F-CTF-CN; (b) N_2 adsorption and desorption isotherms (77 K) curves of F-CTF-CN.

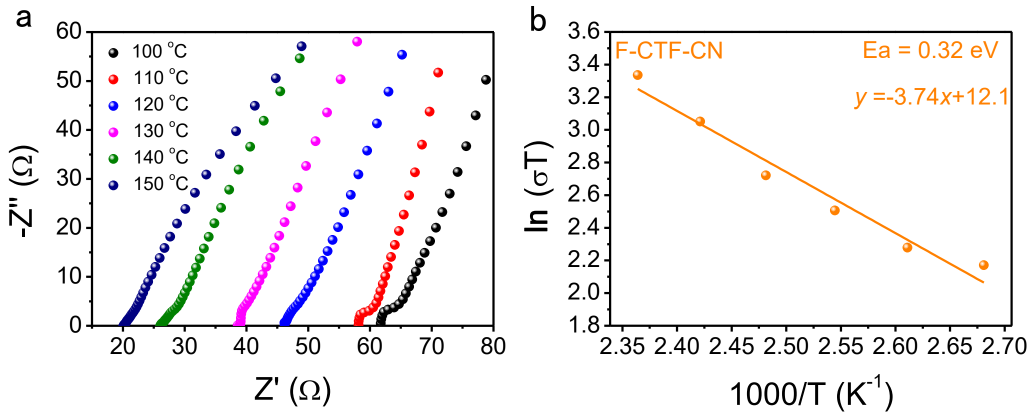

Supplementary Fig. 11. Proton conductivity of F-CTF-CN. (a) Nyquist plots of H3PO4@ F-CTF-CN; (b) Arrhenius plots for H_3PO_4@ F-CTF-CN.

The H_3PO_4@ F-CTF-CN exhibits proton conductivities of 2.35×10^{-2}, 2.55×10^{-2}, 3.12×10^{-2}, 3.77×10^{-2}, 5.21×10^{-2}, and 6.64×10^{-2} S cm^{-1} at 100, 110, 120, 130, 140 and 150 °C, respectively.

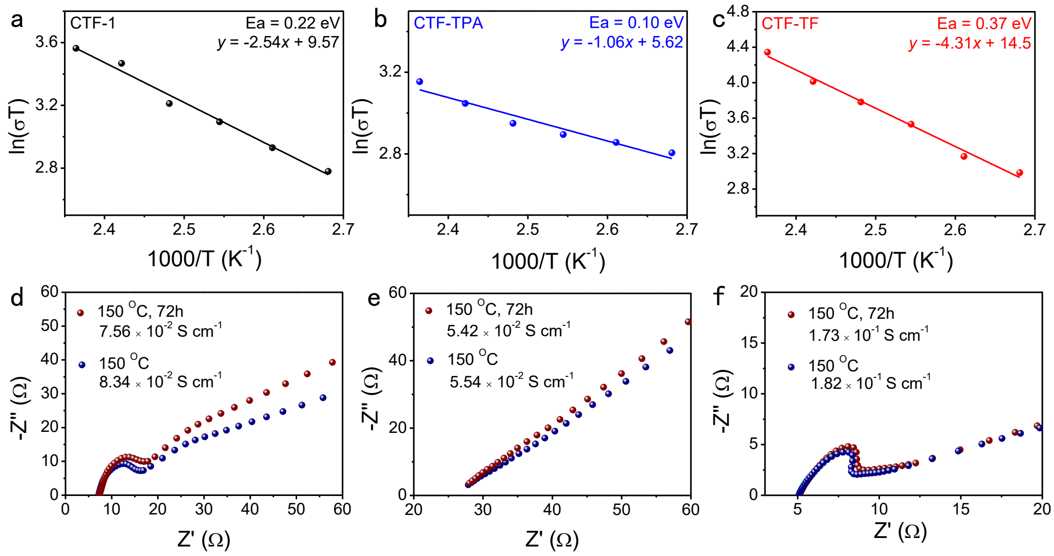

Supplementary Fig. 12. Arrhenius plots for (a) H_3PO_4@CTF-1, (b) H_3PO_4@CTF-TPA, and (c) H_3PO_4@CTF-TF; Proton conductivities of (d) H_3PO_4@CTF-1, (e) H_3PO_4@CTF-TPA, and (f) H_3PO_4@CTF-TF measured at 150 °C after 72h.

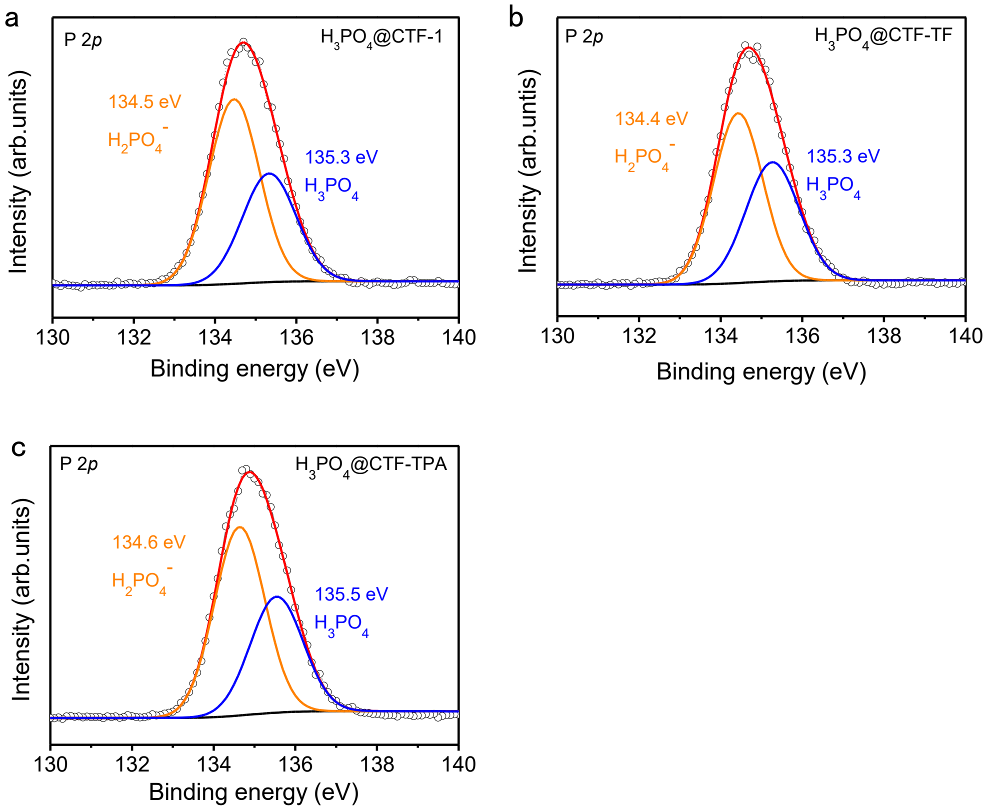

Supplementary Fig. 13. High-resolution P 2*p* XPS spectra of (a) H_3PO_4@CTF-1, (b) H_3PO_4@CTF-TPA, and (c) H_3PO_4@CTF-TF.

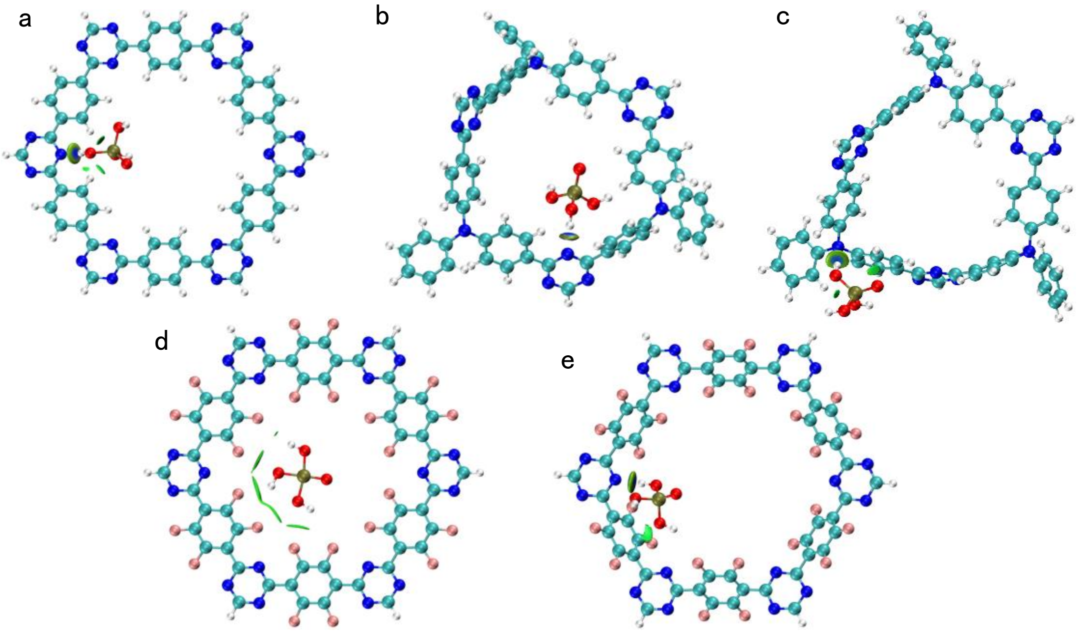

Supplementary Fig. 14. Graphic representations of the binding energy of H_3PO_4 to various interaction sites for CTFs. (a) CTF-1(Triazine N), (b) CTF-TPA (Triazine N), (c) CTF-TPA (Triphenylamine N), (d) CTF-TF (F) and (e) CTF-TF (Triazine N). (H, white; P, brown; O, red; N, blue; C, cyan; F, LT Magenta).

The N(triazine)···H···O hydrogen bonding between CTF-1 and H_3PO_4 yielded a binding energy of -16.150 kcal mol^{-1}. The N(triazine)···H···O hydrogen bonding between CTF-TF and H_3PO_4 yielded a binding energy of -18.767 kcal mol^{-1}, which is higher than that of the CTF-1, indicating the introducing of F can enhance the interaction. The binding energy of hydrogen bonding of CTF-TPA between N(triazine) and H_3PO_4 is -22.347 kcal mol^{-1}. The binding energy for N(triphenylamine)···H···O in CTF-TPA is only -13.767 kcal mol^{-1}, probably due to larger steric effect here that prevents the phosphoric acid molecule from being close to the sites. Considering the amount of hydrogen acceptors (N) in the frameworks, the overall strength of the interaction between the CTF-TPA skeleton and H_3PO_4 should be smaller than CTF-1. Although the covalently bonded fluorine are weaker hydrogen-bonding acceptors as compared to N atoms[1, 2], the isosurfaces show that the introducing of fluorine atoms further increases the intermolecular interactions between the H_3PO_4 and triazine N sites. Moreover, the F atom sites can serve as additional anchoring sites for phosphoric acid, and contribute to the construction of hydrogen-bonding networks for efficient proton conduction.

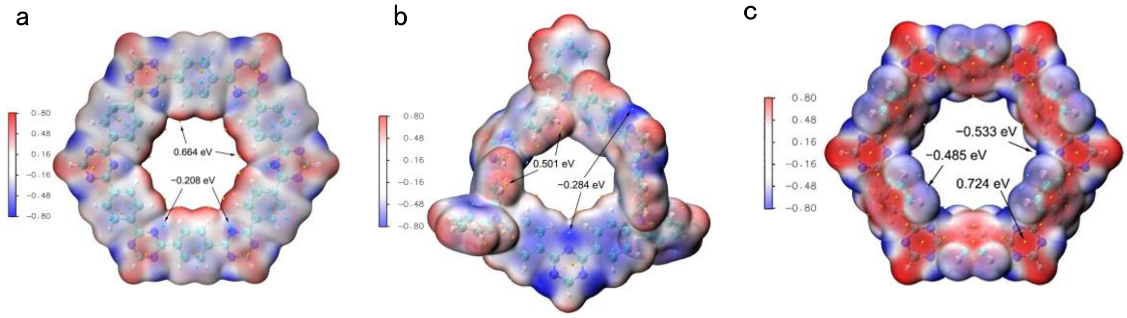

Supplementary Fig. 15. Electrostatic potential of (a) CTF-1, (b) CTF-TPA, and (c) CTF-TF.

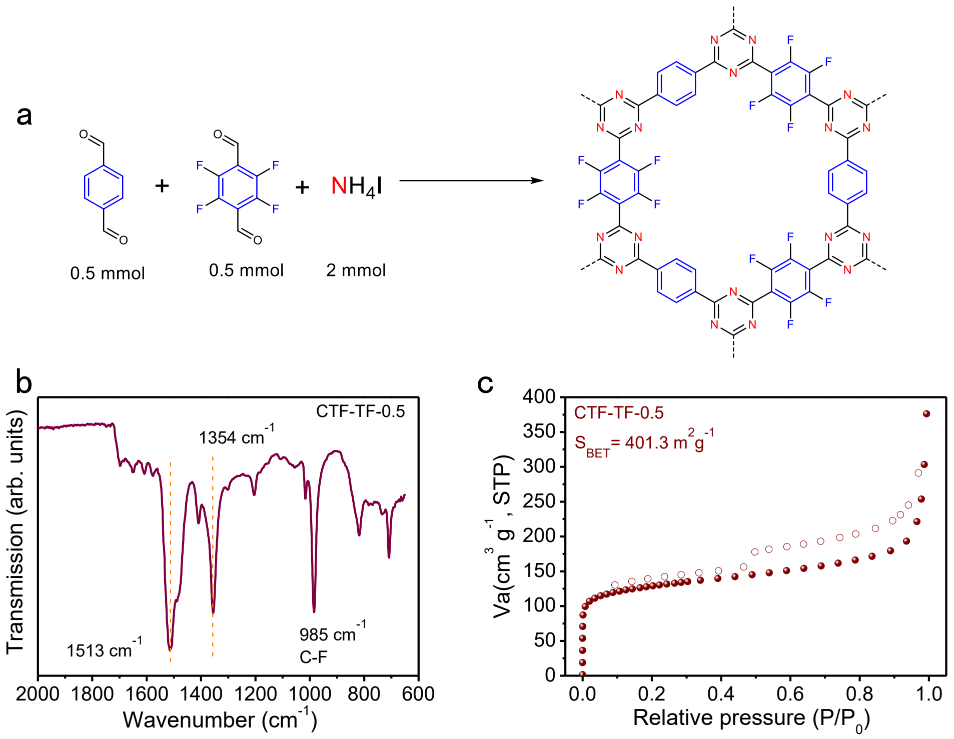

Supplementary Fig. 16. (a) synthesis of CTF-TF-0.5; (b) FT-IR spectra of CTF-TF-0.5; (c) N_2 adsorption and desorption isotherms (77 K) curves of CTF-TF-0.5.

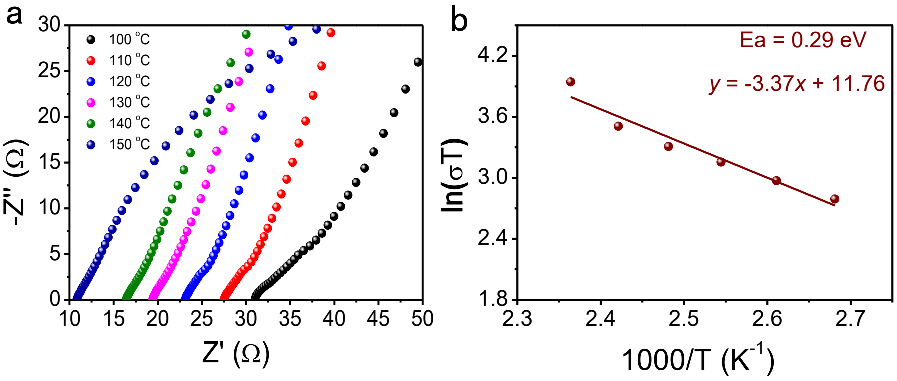

Supplementary Fig. 17. Proton conductivity of CTF-TF-0.5. (a) Nyquist plots of H_3PO_4@ CTF-TF-0.5; (b) Arrhenius plots for H_3PO_4@ CTF-TF-0.5.

The H_3PO_4@ CTF-TF-0.5 exhibits proton conductivities of 4.37×10^{-2}, 5.10×10^{-2}, 5.97×10^{-2}, 6.78×10^{-2}, 8.08×10^{-2}, and 1.22×10^{-1} S cm^{-1} at 100, 110, 120, 130, 140 and 150 °C, respectively.

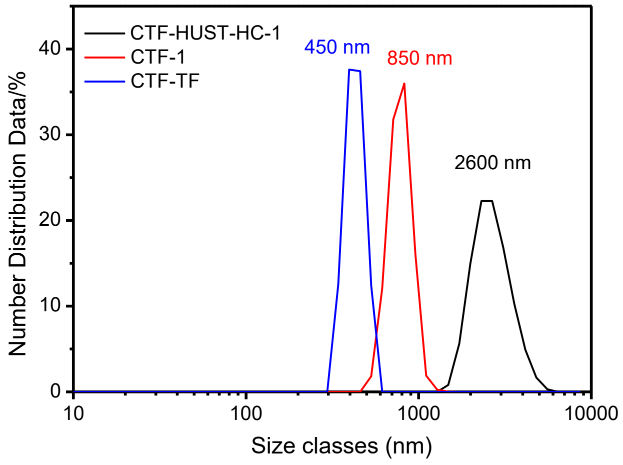

Supplementary Fig. 18. Dynamic light scattering (DLS) experiments of CTF-HUST-HC-1 (wine curve), CTF-1 (black curve) and CTF-TF (red curve) in methanol. The reported CTF-HUST-HC1 synthesized by amidine based condensation was used as a contrast, the average particle size of that is the maximum at 2600 nm.

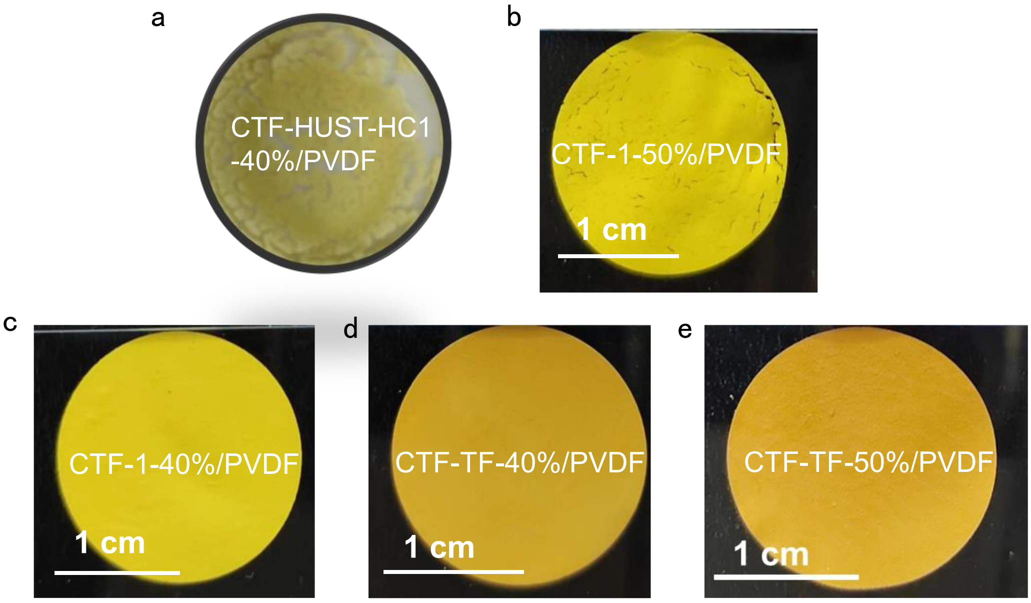

Supplementary Fig. 19. Photograph of membranes (a) CTF-HUST-HC1-40%/PVDF; (b) CTF-1-50%/PVDF; (c) CTF-1-40%/PVDF; (d) CTF-TF-40%/PVDF; (e) CTF-TF-50%/PVDF. CTF-HUST-HC1 has been reported to have high proton conductivity (1.6×10^{-1} S cm^{-1} at 150 °C)[3,4]. When the loading of CTF-HUST-HC1 was 40wt%, obvious cracks was observed, suggesting that the preparation of the membrane failed due to the large particle size.

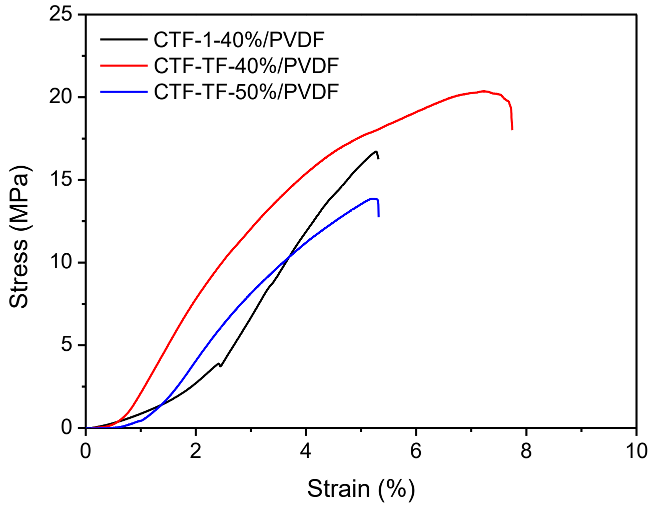

Supplementary Fig. 20. Mechanical strength measurement: Stress–strain curves of MMMs

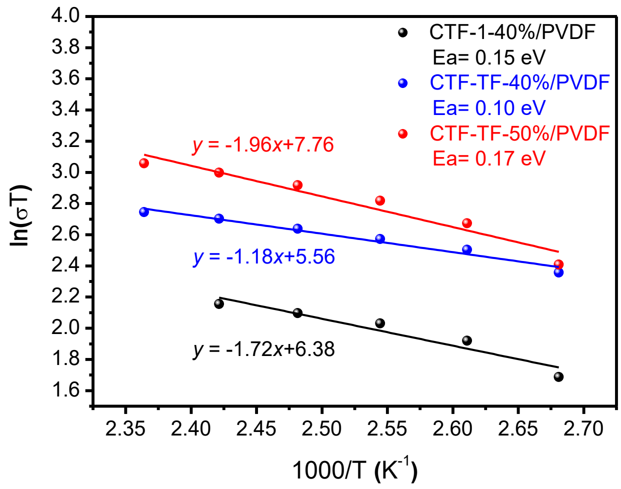

Supplementary Fig. 21. Arrhenius plots for CTF-1-40%/PVDF (black curve), CTF-TF-40%/PVDF (blue curve) and CTF-TF-50%/PVDF (red curve).

Supplementary Table 1.

Comparison of previous reported fluorinated CTFs prepared by different methods with this work.

Monomer	Methods	Conditions	F Contents (wt %)	Surface area (m^2 g^{-1})	References
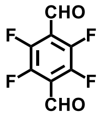	NH_4I-involved method	$Fe(OH)(OAc)_2$; < 160°C; Open system	30.2	407.7	This Work
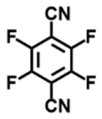	Ionothermal strategy	$ZnCl_2$, 400 °C	23.8	623	Energy Environ. Sci. **2013**, 6, 3684-369
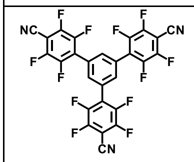	Ionothermal strategy	$ZnCl_2$, 600°C	3.7	1558	J. Mater. Chem. A, **2019**, 7, 17277-17282
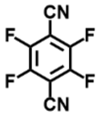	Superacid-catalysis	CF_3SO_3H, 250°C, sealed system; 350°C, N_2	------	2	J. Am. Chem. Soc. **2020**, 142, 6856–6860
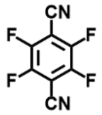	ionothermal strategy	$[Zn(NTf_2)_2]$, 275 °C sealed system	31	367	Angew. Chem. Int. Ed. **2021**, 60, 25688 –256
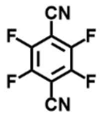	ionothermal strategy	$ZnCl_2$, 400 °C	3.3	638	Sep. Purif. Technol. **2022**, 290, 120857

Supplementary Table 2.

Synthesis of the CTF-1 under variable conditions.

Catalyst	Solvent	Temperature (°C)	Reaction time（h）	Yield [%]	S_{BET} [m^2/g]
$FeCl_3$	Toluene	120	48	46	25
$FeCl_3$	Mesitylene	120	48	33	31
$FeCl_3$	*o*-DCB	120	48	61	42
$FeCl_3$	DMSO	120	48	0	---
$FeCl_3$	DMF	120	48	0	---
$Fe(NO_3)_3$	*o*-DCB	120	48	0	---
$Fe(CF_3SO_3)_3$	*o*-DCB	120	48	50	48
$Fe_2(SO_4)_3$	*o*-DCB	120	48	27	25
$Fe(OH)(OAc)_2$	*o*-DCB	120	48	60	72
$Cu(OAc)_2$	*o*-DCB	120	48	15	8
$Fe(OH)(OAc)_2$	*o*-DCB	40	72	0	---
$Fe(OH)(OAc)_2$	*o*-DCB	80	72	0	---
$Fe(OH)(OAc)_2$	*o*-DCB	40°C, 24 h; 80°C, 24 h; 120°C, 24 h;		66	56
$Fe(OH)(OAc)_2$	*o*-DCB	80°C, 24 h; 100°C, 24 h; 160°C, 24 h		76	100
$Fe(OH)(OAc)_2$	*o*-DCB	40°C, 24 h; 80°C, 24 h; 120°C, 24 h; 160°C, 24 h		88	326.7

Supplementary Table 3.

Elemental contents of CTFs measured by EA.

Sample	Methods	N [%]	C [%]	H [%]	F[%]
CTF-1	Theoretical (wt%)	22.11	75.79	2.1	0.00
	Contents (wt%)	16.32	67.62	4.782	-
CTF-TPA	Theoretical (wt%)	17.49	78.73	3.78	0.00
	Contents (wt%)	12.54	73.55	6.105	-
CTF-TF	Theoretical (wt%)	14.00	48.02	0.00	37.98
	Contents (wt%)	11.96	55.2	-	30.2[a]

a: Determined by XPS. The fluorine contents of CTF-TF-0.5 and F-CTF-CN are 18.92 wt% and 11.76 wt%, respectively, measured by XPS.

Supplementary Table 4.

The proton conductivity of CTFs with the H_3PO_4 loading at different temperatures.

Sample	Temperature (°C)	Proton Conductivity (S cm^{-1})	Activation energy (eV)
H_3PO_4@ CTF-1	100	4.32×10^{-2}	0.22
	110	4.89×10^{-2}	
	120	5.62×10^{-2}	
	130	6.16×10^{-2}	
	140	7.76×10^{-2}	
	150	8.34×10^{-2}	
H_3PO_4@ CTF-TPA	100	4.43×10^{-2}	0.10
	110	4.54×10^{-2}	
	120	4.60×10^{-2}	
	130	4.74×10^{-2}	
	140	5.10×10^{-2}	
	150	5.54×10^{-2}	
H_3PO_4@ CTF-TF	100	0.53×10^{-1}	0.37
	110	0.62×10^{-1}	
	120	0.87×10^{-1}	
	130	1.09×10^{-1}	
	140	1.34×10^{-1}	
	150	1.82×10^{-1}	

Supplementary Table 5.

Comparison of the proton conductivities of previous reported porous organic polymers with this work at high temperatures (100 ℃~200 ℃).

Materials	σ (S cm^{-1})	T (°C)	E_a (eV)	References
H_3PO_4@CTF-1	8.32×10^{-2}	150	0.22	This work
H_3PO_4@CTF-TPA	5.54×10^{-2}	150	0.10	
H_3PO_4@CTF-TF	1.82×10^{-1}	150	0.37	
H_3PO_4@NKCOF-54	2.33×10^{-2}	160	0.29	Angew. Chem. Int. Ed. **2023**, 62, e202217240
H_3PO_4@PBI-COF	1.57×10^{-1}	160	0.24	ACS Materials Lett. **2022**, 4, 2597–2603
H@PTP-COF	1.27×10^{-2}	160	0.17	Angew. Chem. Int. Ed. **2022**, 61, e202208086
H_3PO_4@CTF−H	1.6×10^{-1}	150	0.248	ChemSusChem **2022**, 15, e202201298
H_3PO_4@TPB-DABI-COF	1.52×10^{-1}	160	0.17	Angew. Chem. Int. Ed. **2021**, 60, 12918
im@XJCOF-1	4.38×10^{-2}	140	0.21	ACS Nano **2021**, 15, 19743
H_3PO_4@TPB-DMeTPCOF	1.91×10^{-1}	160	0.34	Nat. Commun. **2020**, 11, 1981.
H_3PO_4@COF-F6	4.2×10^{-2}	140	0.16	J. Am. Chem. Soc. **2020**, 142, 14357
PA@EB-COF	2.77×10^{-2}	180	0.35	J. Mater. Chem. A, **2020**, 8, 13702
im@TPB-DMTP-COF	4.37×10^{-3}	130	0.38	Nat. Mater. **2016**, 15, 722
CTF-1-40%/PVDF (Membrane)	2.07×10^{-2}	150	0.15	This work
CTF-TF-40%/PVDF (Membrane)	3.68×10^{-2}	150	0.10	
CTF-TF-50%/PVDF (Membrane)	5.03×10^{-2}	150	0.17	

Supplementary Table 6.

Summary of the binding energy and proton dissociation energy results of the theoretical calculation (PA:H_3PO_4).

	CTF-TF+PA (Triazine N…H)	CTF-TF+PA (F…H)	CTF-1+PA (Triazine N…H)	CTF-TPA+PA (Triphenyl-amine N…H)	CTF-TPA+PA (Triazine N…H)
Hirshfeld partition (IGMH)	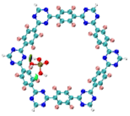	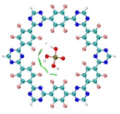	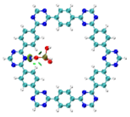	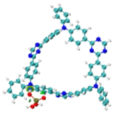	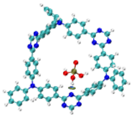
Proton dissociation enthalpy (eV)	4.481	4.265	4.543	4.515	4.496
Binding energy (kcal/mol)	-18.767	-5.328	-16.150	-13.767	-22.347

Supplementary Table 7.

Mechanic strength analysis results from the stress–strain curves.

Samples	Young's modulus (MPa)	Ultimate Stress (MPa)	Strain at break (%)
CTF-1-40%/PVDF	386.23	16.6	5.3
CTF-TF-40%/PVDF	401.26	20.3	8.2
CTF-TF-50%/PVDF	327.35	13.8	5.2

Supplementary Table 8.

The proton conductivity of CTFs based membranes at different temperatures.

Sample	Temperature (°C)	Proton Conductivity ($S\ cm^{-1}$)	Activation energy (eV)
CTF-1-40%/PVDF	100	1.45×10^{-2}	0.15
	110	1.78×10^{-2}	
	120	1.94×10^{-2}	
	130	2.02×10^{-2}	
	140	2.09×10^{-2}	
	150	2.07×10^{-2}	
CTF-TF-40%/PVDF	100	2.83×10^{-2}	0.10
	110	3.19×10^{-2}	
	120	3.33×10^{-2}	
	130	3.47×10^{-2}	
	140	3.61×10^{-2}	
	150	3.68×10^{-2}	
CTF-TF-50%/PVDF	100	2.98×10^{-2}	0.17
	110	3.78×10^{-2}	
	120	4.26×10^{-2}	
	130	4.59×10^{-2}	
	140	4.85×10^{-2}	
	150	5.03×10^{-2}	

Supplementary references:

1. C. Dalvit & A. Vulpetti. Weak intermolecular hydrogen bonds with fluorine: detection and implications for enzymatic/chemical reactions, chemical properties, and ligand/protein fluorine NMR screening. *Chem. Eur. J.* **22**,7592–760 (2016).

2. D. O'Hagan. Understanding organofluorine chemistry. An introduction to the C–F bond. *Chem. Soc. Rev*. **37**, 308-319 (2008).

3. Liu, M. Y. et al. Controlling monomer feeding rate to achieve highly crystalline covalent triazine frameworks. *Adv. Mater*. **31**, e1807865 (2019).

4. Liu, M. Y. et al. Hydrogen bond activation by pyridinic nitrogen for the high proton conductivity of covalent triazine framework loaded with H_3PO_4. *ChemSusChem* **15**, e202201298 (2022).
